# Perceptions of Surgeons in the Kurdistan Region of Iraq Regarding the Use of Artificial Intelligence

**DOI:** 10.7759/cureus.64885

**Published:** 2024-07-19

**Authors:** Dawan J Hawezy

**Affiliations:** 1 Surgery, Faculty of General Medicine, Koya University, Koya, IRQ

**Keywords:** perception, general surgery, survey, artificial intelligence, surgeon

## Abstract

Introduction: Artificil intelligence (AI) is revolutionizing healthcare by seamlessly integrating into various aspects of human life. From robotic surgery to virtual nursing assistants and image analysis applications, AI is transforming the way we approach and deliver healthcare. By leveraging AI, patients can gain a deeper understanding of their symptoms, empowering them to make informed decisions about their health and ultimately improving their quality of life.

Methods: An online survey collected data from social media platforms regarding the surgeon society in the Kurdistan region of Iraq. All statistical analyses were carried out using IBM SPSS Statistics for Windows, Version 25 (Released 2017; IBM Corp., Armonk, New York).

Results: A total of 316 surgeons responded to the survey. A significant majority believed that using artificial intelligence benefits patients, and a substantial number advocated for its avoidance as a matter of principle. More than half said that AI would always impact education, and half of the participants said that AI would always affect complication prediction.

Conclusions: This is the first study investigating surgeon attitudes and perceptions regarding the use of AI in the Kurdistan region. The surgeons who responded generally appreciated AI's use in their practice. Notably, general surgeons showed greater openness to integrating AI into their daily practices compared to those in other surgical specialties.

## Introduction

Artificial intelligence (AI) has the potential to disrupt workflows and increase productivity in healthcare significantly, stemming from its ability to amass more experience than any single human could in their lifetime. AI is the ability of a machine to think and learn [1].

The Industrial Revolution saw a significant increase in productivity and standard of living as society incorporated primary machines into human labor. Similarly, we now face technology that not only surpasses its predecessors but also possesses the potential to outsmart its creators, a development that simultaneously amazes and terrifies us. This is where artificial intelligence comes in [2].

AI is revolutionizing healthcare by seamlessly integrating into various aspects of human life. From robotic surgery to virtual nursing assistants and image analysis applications, AI is transforming the way we approach and deliver healthcare. By leveraging AI, patients can gain a deeper understanding of their symptoms, empowering them to make informed decisions about their health and ultimately improving their quality of life [3].

Diagnostic and decision-making errors rank as the second most frequent cause of preventable harm. Making decisions is one of the most complex and essential tasks for trauma surgeons. Time constraints and high cognitive loads from processing a lot of information force them to use mental shortcuts, which can lead to mistakes and preventable patient injury [4].

AI research has made substantial advancements in multiple medical fields, offering the potential to improve medicine by enhancing therapies, forecasting results, and boosting efficiency [5]. Notably, AI-powered diagnostic algorithms have demonstrated exceptional performance in radiology [6], pathology [7], and dermatology [8]. However, their use in the surgical field still needs further improvement. This gap is probably caused by multiple factors, such as the surgical environment, sophisticated human interaction, a perceived lack of necessity, and little understanding and evidence of AI uses in practical surgery [9]. Despite the fact that 93% of surgeons believed that COVID-19 had an impact on surgical procedures [10], the pandemic has led to the widespread use of AI in medical fields due to its numerous advantages. These include its capacity to analyze self-reported data, analyze X-rays, recognize images from computed tomography (CT), and administer medical treatments [11].

## Materials and methods

After receiving ethical approval from the faculty of general medicine at Koya University, this online survey, conducted via Google Forms and distributed from January 7 to January 28, 2024, collected data from social media platforms regarding the surgeon society in the Kurdistan region of Iraq.

We developed the questions after reviewing the current literature. The web-based survey was organized into two sections, consisting of six questions about demographics and five questions about AI knowledge.

First, we asked if the participant had heard of AI. Next, we measured the respondents' agreement with various statements about seven aspects that AI can influence in surgical practice using a five-point Likert scale (1 = never, 2 = rarely, 3 = sometimes, 4 = frequently, and 5 = always). Then, we asked the participants about their expectations of AI and its future in the surgical field, using 11 statements. We checked their agreement using a three-point Likert scale (agree, neutral, and disagree) in two sections. Finally, we asked the participants to write down their thoughts about AI in a brief note. A copy of the Google Form is given in the Appendices.

Statistical analysis

We manually digitalized the completed questionnaires before analysis. We used descriptive statistics to describe the sample based on factors such as age, gender, physician specialty, and type of health sector service. We excluded questionnaires with unanswered or incomplete portions from the study. We predicted that the findings would be statistically significant if the p-values were less than 0.05. We conducted all statistical analyses using IBM SPSS Statistics for Windows, Version 25 (Released 2017; IBM Corp., Armonk, New York).

## Results

A total of 316 surgeons completed the survey (Table 1), including 246 surgeon specialists (77.8%) and 70 surgical residents (22.2%). Of the surgeons, 20 were older than 60 years, 105 were between 20 and 40, and 191 (60.4%) were between 40 and 60. Of the employees, 9.2% (29 participants) were exclusive to non-academic institutions, while 164 surgeons (51.9%) worked at private and academic hospitals. Among the surgeons, 172 (54.4%) were general surgery specialists, followed by ENT, obstetrics and gynecology, and orthopedics. The work titles of 144 (45.6%) participants were academic.

**Table 1:** General information about responders

Item		Number	Percentage
Specialty level	Specialist	246	77.8
	Surgical resident	70	22.2
Age	20-40	105	33.2
	40-60	191	60.4
	More than 60	20	6.3
Gender	Male	248	78.5
	Female	68	21.5
Specialty	General Surgery	172	54.4
	ENT	42	13.3
	Obstetric and Gynecology	25	7.9
	Pediatric Surgery	18	5.7
	Urology	18	5.7
	Orthopedics	19	6
	Plastic Surgery	22	7
Academic title	Academic	144	45.6
	Non-academic	172	54.4
Hospital type	Nonteaching hospital	29	9.2
	Teaching hospital	104	32.9
	Non-teaching/private	19	6
	Teaching/private	164	51.9

Precisely 17 participants (5.4%) expressed that they were unaware of AI. However, most respondents (59.8%, n = 189) claimed to be aware of artificial intelligence, explained it well, or were experts in the field (Table 2).

**Table 2:** Knowledge about AI

Knowledge		Frequency	Percentage	Valid percentage	Cumulative percentage
Valid	No	17	5.4	5.4	5.4
	Yes, and I could explain well what it is about	171	54.1	54.1	59.5
	Yes, and I am an expert in that field.	18	5.7	5.7	65.2
	Yes, but I don't know exactly what it is	110	34.8	34.8	100.0
	Total	316	100.0	100.0	

AI implementation and expectations

The survey revealed a diverse range of perspectives among the participating surgeons. While a significant majority (59%, n = 187) believed that using artificial intelligence benefits patients, a substantial number (43%, n = 138) advocated for its avoidance as a matter of principle. This variety of viewpoints underscores the complexity of the issue and the need for further discussion and research.

**Figure 1:**
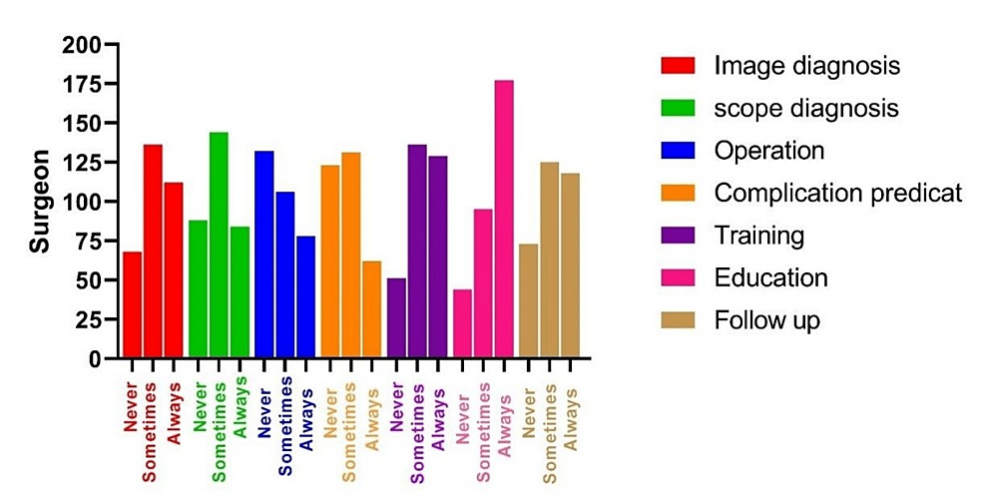
The effect of AI on different aspects of surgical practice Y-axis: number of surgeons.

We selected seven key areas where AI could potentially enhance surgical practice: image diagnosis, scope diagnosis, operation, complication prediction, training, education, and follow-up. Of the 316 respondents, 200 (63%) said that AI would always impact education, and only 158 participants (50%) said that AI would always affect complication prediction. On the other hand, 131 (41%) said that AI would never or rarely improve operations, as shown in Figure 1 and Table 3.

**Table 3:** AI implementation perception by academic and nonacademic surgeons P-values <0.01 are regarded as significant.

Category	Type	Always	Frequently	Never	Rarely	Sometimes	Total	P-value
Image diagnosis	Academic	8	54	4	16	62	144	0.12
	Non-academic	10	40	8	40	74	172	
Scope diagnosis	Academic	10	37	2	17	78	144	<0.01
	Non-academic	4	36	19	49	64	172	
Operation	Academic	9	43	15	20	57	144	<0.01
	Non-academic	2	23	32	64	51	172	
Complication	Academic	5	29	14	30	66	144	0.134
	Non-academic	2	25	19	54	72	172	
Training	Academic	10	53	4	13	64	144	0.160
	Non-academic	17	47	8	27	73	172	
Education	Academic	27	56	2	14	45	144	0.17
	Non-academic	29	64	6	22	51	172	
Follow-up	Academic	13	53	6	16	56	144	0.682
	Non-academic	10	45	22	28	67	172	

AI-related risks and concerns

The majority of respondents (44%, n = 141) said that AI would ruin the patient-doctor relationship. Only 65 (20%) agreed that robotic surgery could be done on their first-degree relatives. Nearly half of the respondents (45%, n = 144) were afraid of technical data loss. Additionally, 69 participants (21%) did not believe that AI would cause legal problems (Table 4).

**Table 4:** Risks and concerns of AI

Risks and concerns of AI	Agree, N (%)	Neutral, N (%)	Disagree, N (%)
Robotic surgery to be used for yourself or your first-degree relatives	65 (20.5%)	169 (53.4%)	82 (26%)
Fear of technical data loss	144 (45.5%)	147 (46.5%)	25 (8%)
Legal problems	71(22.4%)	176(55.6%)	69(22%)
Destroy patient-doctor relationship	141 (44.6%)	125 (39.5%)	50 (15.8%)

**Figure 2:**
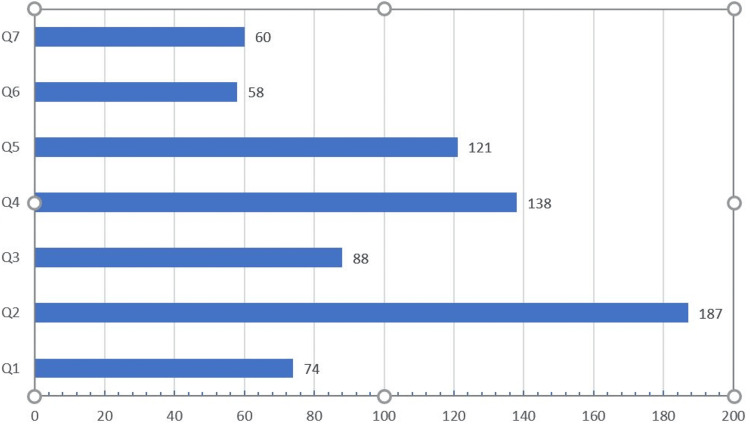
How surgeons perceive and receive AI Q1 In the future, doctors' involvement in patient therapy will be less significant. Q2 In my opinion, using artificial intelligence benefits patients. Q3 AI applications will reduce future treatment errors. Q4 Overall, we shouldn't use AI in medicine. Q5 Physicians tend to depend too much on computers. Q6 Artificial intelligence's impact on medical care is worrying. Q7 Doctors will not fight as hard for a patient's life if AI indicates that their chances of survival are low.

When asked in the study survey, most of the respondents stated that the artificial intelligence approach in the future would help patients receive benefits and navigate the medical process quickly and efficiently. However, they also noted that the effect of artificial intelligence on scars weakens the relationship to medical treatment, as shown in Figure 2.

Respondents provided additional remarks about AI and ML. Here are a few direct quotes from the participants: "I think that innovations in surgery, like robot surgery, make more complex surgical procedures easier, decrease demand on the surgeon's body, and decrease the time of hospital stay." "It can facilitate our work by answering complex questions, demonstrating surgical procedures, and predicting possible complications." "It's a promising advancement, but it can't completely replace surgical branches and hand skills."

## Discussion

Our study provides some early insights into how surgeons and surgical trainees perceive the future application of artificial intelligence in the operating room, directly reflecting the significance of AI in the surgical sector in the Kurdistan area. Age groups, gender, and hospital type do not significantly differ in their perceptions of AI and its future aspects.

Furthermore, more than half of surgeon respondents (189 people, or 59.8%) reported that they knew about AI and were experts; these findings are similar to those from recent studies [4,12]. While some research revealed that less than 50% know AI [9,13], these differences may be due to time, as AI is new. Researchers conducted these three surveys two years ago.

Additionally, 78% (246 participants) and 72% (227 participants) believed that artificial intelligence (AI) could be utilized for image and scope diagnosis, respectively. This aligns with the fact that AI has already begun to play a diagnostic role in the diagnosis of acute appendicitis, the most common surgical disease globally [14]. The thought is divided into academic and non-academic surgeons; academic holders have more prediction than non-academic surgeons regarding image and scope diagnosis. These findings align with [9], where most participants agreed that AI benefits patients. Academic and non-academic surgeons' responses exhibited significant differences, mirroring those of other respondents. Academic surgeons believe that AI contributes to scope diagnosis and operation (p-value less than 0.001), and they acknowledge the possibility of robotic surgery for themselves or their relatives. Only 47 people out of 316 (14%) agreed that gallbladder surgery should be performed by a fully autonomous robot controlled by AI in the future, which is nearly the same as other articles [9].

Our survey showed that only 73 participants (23%) feared losing their jobs. Al-Medfa, Al-Ansari, Darwish, Qreeballa, and Jahrami [15] attribute surgeons' lack of fear of job loss to the nature of their profession; for instance, most vascular surgeons exhibit no fear of job loss. This is similar to the situation in general healthcare, where most surgeons do not fear job loss [1,12,15,16]. However, it is essential to note that this fear is specific to specialists, as other specialties may experience fear of job loss [17,18], unlike radiologists [18].

Our study identified seven potential areas where AI could be beneficial in surgical practice. The majority of participants anticipate its application in surgical education, half anticipate its occasional or constant use in surgical diagnosis, including both image and scope diagnosis, and only a minority believe it could replace human surgical management. All of these results align closely with previous research [9,13,16]. However, our respondents differ from others in their belief that AI could potentially reduce complications, with the majority not accepting this notion, whereas most general surgeons do [9].

Only a minority of participants agree that AI can be used in complication prediction. This may be due to a misunderstanding of the details of AI uses. AI can be used in complication prediction, as this study suggests that deep learning can identify safe and dangerous zones of dissection and other anatomical structures in the surgical field during laparoscopic cholecystectomy with a high degree of performance. As additional evidence emerges on the safety and effectiveness of using AI in the operating room, these automated computer vision tasks have the potential to augment performance and eventually be used for real-time decision support and other quality-improvement initiatives in the future [19].

Only 69 surgeons (22%) of those who answered the survey agreed that ethical and legal concerns about using AI differ from what was found in [13,17], which showed that 62% (195 participants) and 57% (180 participants) of people often said these concerns were obstacles to using AI in real life. Moreover, based on the responses, we found that 142 surgeons (45%) fear technical issues such as hacking, whereas in [9], only 15% are concerned about data privacy and its loss, and only 6% are familiar with the specifics of data privacy in [20].

We analyzed the responses of seven different surgical specialists separately and found significant differences in their perspectives on the role of AI in improving surgical practice (p-value less than 0.001). General surgeons, more than other specialists, concur with the idea that AI can enhance operations; they also agree more than other surgical specialists that AI can influence surgical practice through training (p-value = 0.009), education (p-value = 0.002), and follow-up (p-value = 0.032). The results from general surgeons closely align with those of other studies [9,20]. On the other hand, urological surgeons agree more than other surgical specialists about performing surgery using AI tools like robotic surgery, followed by general surgeons (33.3% and 22.7%, respectively, with a p-value of 0.19).

A few of our study's limitations are noteworthy. The first is that there need to be more participants, especially in the subspecialties. Second, the questions were closed-ended, so we could not ask open-ended inquiries. Third, selection bias could be present. Survey participants may have been more enthusiastic and optimistic than non-participants.

## Conclusions

This is the first study investigating surgeon attitudes and perceptions regarding the use of AI in the Kurdistan region. The surgeons who responded generally appreciated AI's use in their practice. Notably, general surgeons showed greater openness to integrating AI into their daily practices compared to those of other surgical specialties. Most of them support using AI in various management procedures and are unconcerned about losing their employment. Surgeons understand the various applications of AI in diagnosis, treatment of surgical problems, AI-powered surgical research, training, and education. There are notable distinctions between the perspectives of academic and non-academic surgeons regarding legal and technological matters. Surgeons who are apprehensive about the future may require additional explanations and assistance.

## Figures and Tables

**Figure 3:**
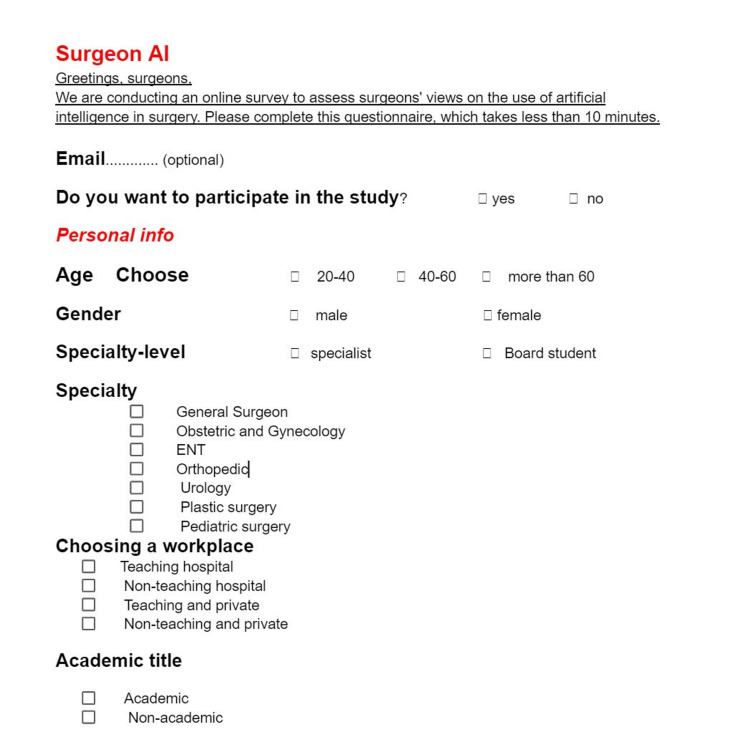
Questionnaire about the perception of surgeons in the Kurdistan region regarding the use of artificial intelligence (Part 1)

**Figure 4:**
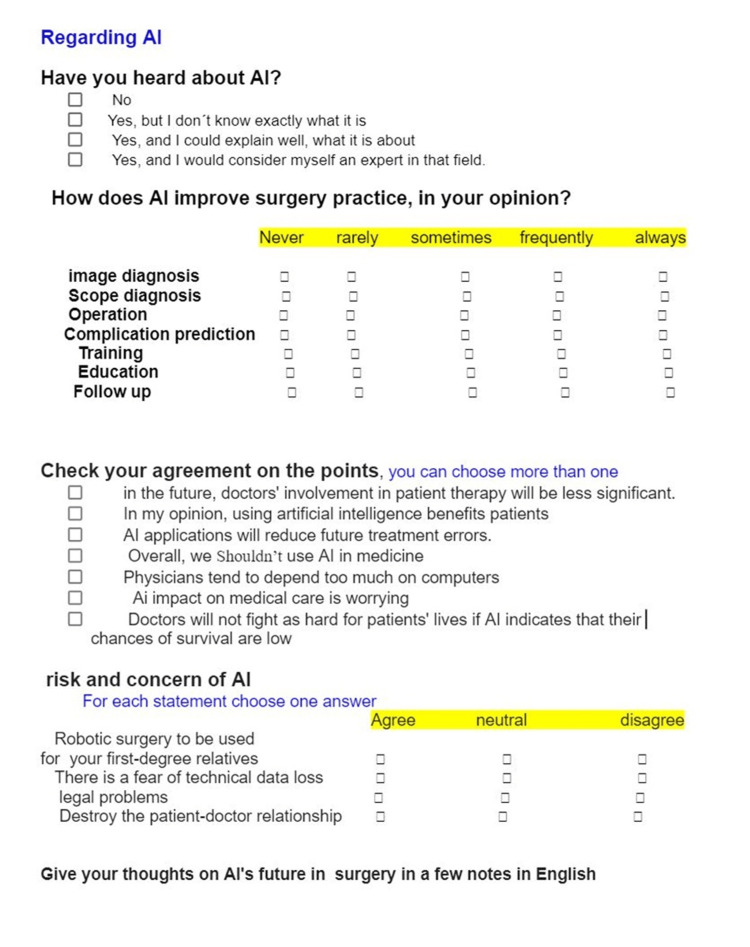
Questionnaire about the perception of surgeons in the Kurdistan region regarding the use of artificial intelligence (Part 2)

## Appendices

A questionnaire about the perception of surgeons in the Kurdistan region regarding the use of artificial intelligence is given in Figures 3, 4.
